# Influence of Temperature, Strain Rate, and Condition on the Mechanical Response of an AlSi-PES Abradable

**DOI:** 10.1007/s11340-025-01211-z

**Published:** 2025-07-01

**Authors:** R. Lye, A. Pellegrino, C. Bennett, J. Rouse, P. Agyakwa, G. Zumpano

**Affiliations:** 1 Mechanical and Aerospace Systems Research Group, Faculty of Engineering, University of Nottingham, Nottingham, UK; 2 Department of Mechanical Engineering, University of Bath, Bath, UK; 3 Department of Electrical and Electronic Engineering, University of Nottingham, Nottingham, UK; 4 Rolls-Royce plc, P.O. box 31, DE24 8BJ Derby, UK

**Keywords:** Gas turbine, Thermal analysis, Abradable, Temperature dependence, Strain rate dependence, Failure behaviour

## Abstract

**Background:**

To improve the efficiency and operational stability of aero-engine compressors, abradable liners are used to facilitate reduced clearances between the blade tips and the surrounding casing. However, their properties are highly variable due to sensitivities in the plasma spraying process and complex in-service phenomena such as blade-casing interactions and thermal ageing. The abradable variability makes it difficult to model blade-casing interactions accurately, leading to suboptimal blade geometries and clearances.

**Objective:**

This study addresses the impact of abradable condition on its mechanical behaviour and on the blade-casing interaction response.

**Methods:**

The response and failure behaviour of an aluminium-silicon-polyester abradable under quasi-static (0.01 s$^{-1}$) and high-rate (850 s$^{-1}$) loading conditions over a range of temperatures and pre-treatments have been characterised. Pre-treatments representative of various points throughout the lifecycle of an abradable were used.

**Results:**

The abradable exhibited sensitivity to strain rate, temperature, and particularly the state of the polyester phase. Ageing the polyester reduced its compliance, increasing the failure stress by up to 10% at high loading rates compared to the as-sprayed material. In the compacted specimens, ageing increased the failure stress by up to 50%, attributed to enhanced thermal stability from increased polyester crystallinity.

**Conclusions:**

A better understanding of abradables and their failure behaviour will improve compressor blade and abradable system design, enabling optimal tip clearances and enhancing overall engine performance. These tests provided an account of condition-specific compressive failure behaviour, beginning to bridge the gap between phenomenological accounts from experimental blade–abradable rub tests and observed abradable response.

## Introduction

To reduce aerospace CO$_2$ and NO$_{\text{ x }}$ emissions in accordance with Flightpath 2050 and the Clean Aviation programmes [1, 2], aero-engine manufacturers are exploring ways to increase the overall pressure ratio (OPR) and thermal efficiency of their gas turbines [3]. The efficiency of gas turbines is largely driven by core engine systems including the compressor, combustor, and turbine, with significant attention directed towards minimising clearance between compressor blade tips and surrounding casing to prevent high pressure air flowing upstream. This inevitably leads to more frequent blade-casing interactions, requiring sacrificial abradable liners that ideally wear preferentially to the blades, and are typically atmospherically sprayed onto internal casing surfaces. These abradables comprise a metal matrix such as aluminium-silicon (AlSi), a dislocator phase such as polyester (PES) or hexagonal boron nitride (hBN), and porosities. Within the compressor AlSi-PES abradables are commonly used with titanium blades up to temperatures of $345^\circ$C [4]. While abradables enable the minimal blade tip clearances necessary for optimal compressor efficiency, their properties are highly variable, being dependent on manufacturing processing conditions and service history [5–8].

Studies have highlighted how specific abradable types within acceptable Rockwell hardness bounds can cause different blade responses [5], with abradables of different hardnesses evolving differently during blade casing rub events. Comprehensive studies at the University of Sheffield using a simplified test rig with stroboscopic imaging capabilities gathered blade images after each rub to investigate the influence of blade tip velocity, incursion rate, abradable type, and hardness on the interaction mechanisms [6, 8–13]. These investigations found that high hardness abradables led to excessive blade wear while low hardness abradables caused abradable adhesion to the blade tip. Differences in mechanism across the blade chord length were also observed, attributed to local variations in constituent phase deposition, with dislocator phases acting as barriers to thermal diffusion [14]. However, these investigations were largely phenomenological and did not consider the evolution of constituent phases. Martinet et al. [15] performed tests using a projectile-based apparatus with abradable temperatures ranging from room temperature to $270^\circ$C for relative tip speeds of 125 m$\cdot$s$^{-1}$ and average incursion depth of 100 µm. Tests at room temperature resulted in clean cuts with smooth surfaces and opened porosities, while at high temperatures, the polyester phase softened and prevented clean cutting of metal particles, increasing friction between the abradable projectile and cutting tool.

Investigations into blade response during blade-casing interaction events have employed novel test rigs to understand the complex dynamics involved. TU Dresden developed a test rig using representative part geometries to investigate the influence of rubbing frequency per revolution and abradable condition on blade response [16, 17], identifying the number of interactions per revolution and abradable evolution as causes for observed changes in blade response. Similarly, Ohio State University developed a test rig accepting representative blades with a curved $90^\circ$ liner segment, conducting tests with bare metal casing, abradable lined casing, and ceramic matrix composite blades [18–21]. Results showed that abradable liners significantly reduced contact forces compared to bare steel casings, though the abradable material compacted after multiple rubs, leading to increased forces. The use of complex blade geometries meant that plastic deformation rather than abradable wear was observed, which is rarely seen in other works that often use heavily simplified thick rectangular blades with much higher stiffness than true engine blades [6, 8–13].

Several studies have aimed to determine the constitutive properties of abradables through various experimental and modelling approaches. A common modelling method involves creating two-dimensional models based on material micrographs to estimate elastic properties of abradables with varying PES contents [22–25]. In these studies, elastic properties of constituent phases were determined via nano hardness testing or three-point bending tests of sprayed material on substrates. AlSi-PES abradables with 58% PES had a Young’s modulus of 8 GPa, while 72% PES content showed a slight reduction to 7.6 GPa. However, temperature effects and debonding between particles were not considered. Lye et al. [5] used inverse analysis methods to obtain homogenized abradable properties over a range of superficial Rockwell hardnesses, demonstrating significant influence on blade response. Cheng et al. [26] created three-dimensional models from X-ray computed tomography data to model deformation and failure behaviour in representative volume elements. This method used Johnson Cook plasticity for the AlSi phase and Richeton plasticity for the polyester phase, with failure occurring in thin AlSi struts between PES particles and porosities.

**Table 1:** Atmospheric plasma spraying parameters

Stand-off	Step size	Scan speed	Current	Voltage	Primary	Primary gas	Carrier	Powder
[mm]	[mm]	[mm$\cdot \mathrm s^{-1}$]	[A]	[V]	gas	flow rate	gas	feed rate
						[scfh]		[g$$\cdot \mathrm min^{-1}$$]
90	5	1000	900	30	Ar	82	Ar	25 (external)

Experimentally, Pellegrino et al. [7] investigated AlSi-PES abradables over a temperature range in quasi-static tension and compression, additionally using Split-Hopkinson pressure bar (SHPB) for high strain rate compression tests. Large tension-compression asymmetry and strong temperature and strain rate dependencies were observed. Skiba et al. [27, 28] also used SHPB apparatus to characterise high-rate behaviour across strain rates from 10$^{-3}\,\text {s}^{-1}$ to 10$^3\,\text {s}^{-1}$ with temperatures up to $360^\circ$C. A temperature dependence but no strain rate dependence at room temperature was found, leading to the development of a bilinear thermoelastoviscoplastic constitutive law. Chevrier et al. [29] determined properties for standard and modified Johnson-Cook constitutive laws, with the modified model accounting for exponential dependence between strain-rate sensitivity parameter and temperature, performing better at high temperatures and strain-rates. Hopkins [30] and Johnston et al. [31] developed methods to manufacture free-standing tensile specimens by spraying directly into water-soluble moulds made from Aquapour 4015 rather than onto metal substrates. It was found that AlSi-based abradables had extremely low strains to failure and were heavily influenced by constituent phase distributions. Mean strain to failure for AlSi-hBN was approximately 0.2% with mean Young’s modulus of 17 GPa. Their hardness testing study identified up to 75% variations between hardness values from different overhaul facilities for AlSi-hBN abradables, attributed to operator and equipment inconsistencies.

While current literature considers effects of varying constituent phase amounts, temperature dependence, and strain rate dependence, no work to date has considered the preconditioning effects such as compaction or thermal ageing of constituent phases throughout operational life. This work investigates AlSi-PES abradable behaviours in as-sprayed, aged, compacted, and combined aged and compacted states at high strain rates over temperatures from $20^\circ$C to $200^\circ$C as experienced in the intermediate pressure compressor stages, representing the abradable at various lifecycle stages to enable optimised compressor blade designs accounting for property variations during blade-casing interactions.

## Methodology

In this section the sample manufacturing methods, thermal analyses, test procedures, and numerical model set-up are presented. The outcomes of these thermal analyses were then used to determine the abradable conditioning and test parameters.

### Specimen Manufacture and Material

The high-rate behaviour of an AlSi-PES abradable has been investigated over a range of temperatures and with various pre-conditions. These samples were atmospherically plasma sprayed with a Praxair SG-100 spray gun mounted on a robot arm, with the parameters used shown in Table 1. A horizontal raster scan pattern with a vertical spacing of 5 mm and scan speed of 1000 mm$\cdot \mathrm s^{-1}$ were used as they produced an even spray. Typically abradables are sprayed onto a substrate material such as titanium or steel. However, to create free-standing specimens without the need to machine away a substrate material, a water soluble and thermally stable material, Aquapour 4015, was used. When using Aquapour as a substrate it is not necessary to use a bond coat. Once the abradable material was-sprayed, the substrate material was washed away under running water and ground flat on one side. Then cylindrical samples with a diameter of 6 mm were extracted via wire electron discharge machining (EDM). Finally, the individual samples were ground down to a height 3.5 mm ensuring that both faces remained parallel and concentric.

**Fig. 1:**
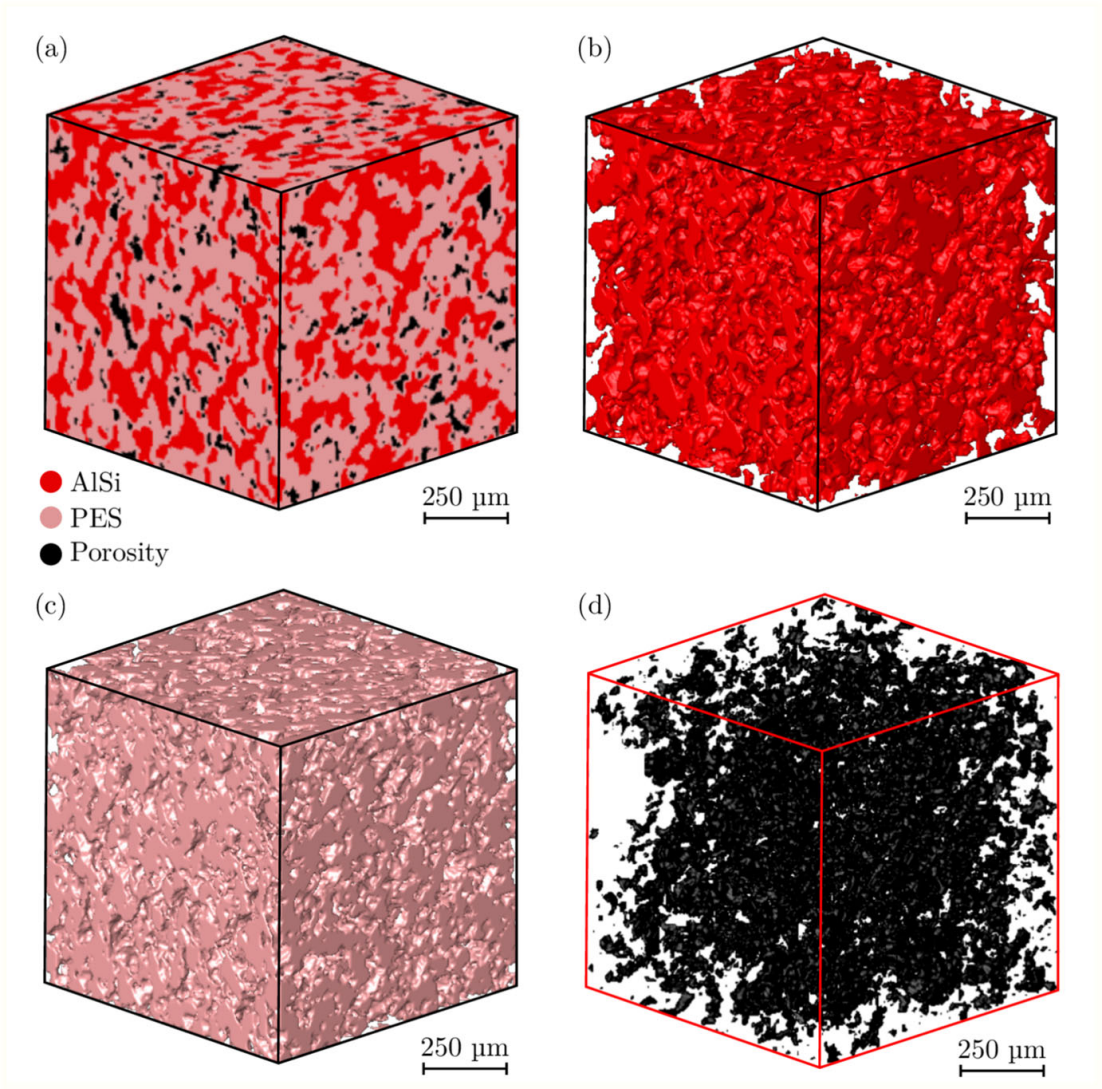
RVE extracted from X-ray CT scan of an as-sprayed sample, with 1 mm edge length. (a) RVE, (b) AlSi phase, (c) PES phase, (d) Porosities

Abradables are commonly characterised by their superficial Rockwell hardness value (HR15Y), which is found using a 12.7 mm ball indenter so that a large and representative volume of material is tested. AlSi-PES abradables typically have an acceptable HR15Y range of approximately 50 to 80, with the abradable samples in this work having an HR15Y value of 65. For a cubic representative volume element the minimum volume has previously been determined to be in the region of 0.1 mm$^{3}$ to 0.33 mm$^{3}$ [26, 28]. Furthermore, all samples were manufactured in a single batch using a simple raster scan pattern on a flat continuos substrate. Hence, the in-batch variability can be assumed to be low as it often arises due to component degradation or feedstock changes [32, 33]. An X-ray computed tomography (XCT) image was obtained from a single as-sprayed specimen using a ZEISS Xradia Versa XRM 500 system, from which a 1 mm$^{3}$ region of interest was extracted, as shown in Fig. 1. The imaging parameters included a beam voltage of 80 kV, a current of approximately 87 µA, and an exposure time of 1.5 s. Scans were conducted using 0.4 times optical magnification. The source and detector were positioned to achieve a spatial resolution of 2.54 µm over an approximate 2.5 mm field of view. Beam filtering was applied to minimise imaging artifacts, and 2x2 camera binning was used during data acquisition. A total of 1601 projection images were collected over a $360^\circ$ rotation, with the datasets being reconstructed using the ZEISS Xradia Reconstructor software. The as-sprayed material comprised 37% AlSi, 58% aromatic semi-crystalline polyester, and 5% porosities.

### Identifying phase transitions

**Fig. 2:**
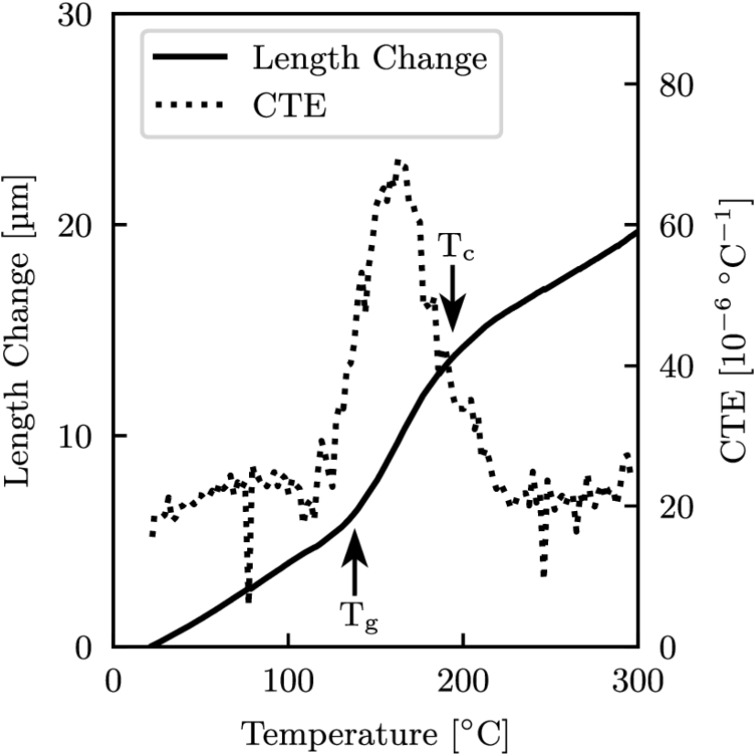
TMA of an as-sprayed specimen from room temperature to $300^\circ$C with a heating rate of $5^\circ$C.min$^{-1}$

Abradables have a wide operating range, an AlSi-PES abradable can be used from ambient air temperatures to $345^\circ$C, and as such, it is important to have an understanding of the transition temperatures of the individual phases over the materials full operating range [4]. To do this a TA Instruments Q400 thermomechanical analyser (TMA), and a TA Instruments differential scanning calorimetry (DSC) 2500 were used. The samples used in these analyses were extracted from the same batch and in the same way as those for the mechanical testing, having a diameter of 4 mm for both methods, and thicknesses of 2.5 mm and 1.5 mm for the TMA and DSC respectively. These smaller specimen sizes enabled better temporal resolution and reduced thermal lag. The TMA tests were conducted with a heating rate of $5^\circ$C$\cdot$min$^{-1}$ from a room temperature of $23^\circ$C to $300^\circ$C, which was $25^\circ$C below the materials upper operational temperature limit to avoid melting, with the resulting TMA data shown in Fig. 2.There were two distinct inflections, the first being the glass transition temperature, $T_g$, which occurred at $138^\circ$C, and the second being the cold crystallisation temperature, $T_{c}$, at $194^\circ$C. These temperatures were determined in accordance with BS ISO 11359-2 [34], where the point of intersection between the lines fitted to each of the linear sections were deemed to be the transition temperature. At $T_g$ the PES phase begins to transition into a rubbery state, which is amorphous and has increasing void volumes with increasing temperature, as seen by the gradient increase between $T_g$ and $T_{c}$, and was also evident by the increase in the linear coefficient of thermal expansion (CTE). Whereas at $T_{c}$ cold crystallisation occurred, with the polyester chains moving closer together and aligning into a dense and ordered lamellar structure.

**Fig. 3:**
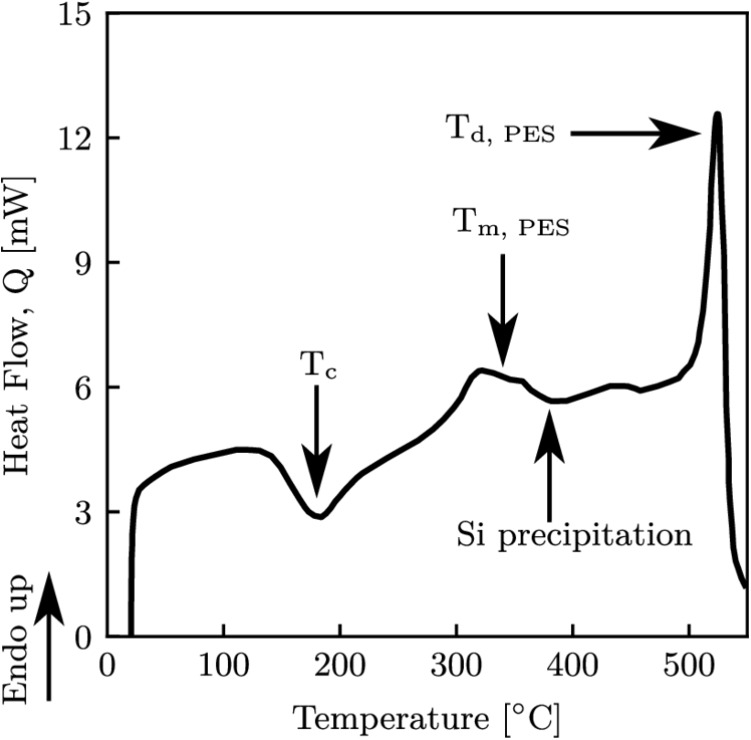
Heat flow plot following DSC analysis for as-sprayed sample

DSC testing was also conducted over a temperature range of $20^\circ$C to $550^\circ$C, with a heating rate of $10^\circ$C.min$^{-1}$, as specified by BS EN ISO 11357-1 [35], with the resulting data shown in Fig. 3. A control test using an empty aluminium sample pan was subtracted from the obtained curve for the as-sprayed abradable material. The first major feature was seen at $190^\circ$C and was attributed to the cold crystallisation of the PES phase, which is characterised by an exothermal peak below the melting point of polymers, and is also in agreement with the TMA findings. PES melting was shown by an endothermic peak beginning at $320^\circ$C, shortly followed by the precipitation of silicon in the AlSi phase [36]. Finally, oxidative decomposition and degradation was seen at $520^\circ$C. The glass transition was not visible, being a second order transformation a step rather than peak would be expected. As the cold crystallisation peak was pronounced, a significant proportion of the PES phase must have been amorphous, as crystalline polymers do not experience any transitions before melting [37]. Therefore, given that $T_g$ was clearly seen in the TMA data, here it was assumed to be overshadowed by the response of the whole material.

### Sample Conditions

Specimens were subjected to pre-treatments so that their condition was representative of an abradable at various points in its lifecycle, from a new abradable as seen during engine commissioning, to one which has been exposed to numerous rub events. The sample conditions investigated were as-sprayed, aged, compacted, and finally aged and compacted. The as-sprayed specimens were quenched as they hit the Aquapour substrate, and as such the PES phase was largely amorphous, with this condition being representative of a newly sprayed abradable. Compacted samples were quasi-statically compressed between two steel anvils to incipient failure at an applied specimen strain of 7%, as measured by contact extensometers, at rate of 0.01 s$^{-1}$. The subsequent level of porosity removal was comparable to an abradable following the initial rubs during running-in, where the material has not been held at an elevated temperature for a prolonged period, but has been subjected to blade rubs. It is possible for abradable material to be held at a high temperature for prolonged periods without experiencing any blade rubs. To represent this scenario, specimens were thermally aged at $200^\circ$C for 100 h with a heating and cooling rate of $5^\circ$C$\cdot$s$^{-1}$, giving ample time for primary and secondary recrystallisation [38, 39]. The AlSi-PES abradable can be used in environments ranging from ambient temperatures to $345^\circ$C [40], and the ageing temperature of $200^\circ$C was chosen as this was where the recrystallisation peak was seen. Finally, some of the specimens were thermally aged and compacted, which is representative of an abradable that is later in its lifecycle.

To determine the influence of thermal ageing on the properties of the constituent phases, Nano-Hardness Testing (NHT) was conducted using a Berkovich indenter. As-sprayed and thermally aged specimens were subjected to a total of 30 indents each, 15 in both the AlSi and PES phases. The initial contact force was set to 0.02 mN, and a loading rate of 1 mN$\cdot s^{-1}$ was applied until a maximum depth of 240 nm was reached, where a dwell time of 5 s was set before load removal. The projected contact area as a function of the indentation depth, $A(h_c)$, is described by

1 $$\begin{aligned} A(h_c) = C_0h_c^2\mathrm , \end{aligned}$$

with $C_0$ being a constant relating to the indenter type [41]. The constant $C_0$ is 24.56 for a perfectly sharp Berkovich indenter, while the one used in the study had a value of 24.482 [42].

The mean reduced moduli, $E_r$, and the standard deviations obtained from the NHT are presented in Table 2. The reduced modulus of the AlSi phase for the as-sprayed and thermally aged specimens have similar mean values and standard deviations. However, the PES phase of the as-sprayed material had a mean reduced modulus of 5.1 GPa, while that of the thermally aged sample was 8.2 GPa and with a larger spread. This increased Young’s modulus was indicative of the ageing process increasing the PES crystallinity. The increased spread is also to be expected, as crystalline regions grow radially forming spherulites, regions of amorphous material will still exist in the areas between tangent spherulites [43, 44]. Furthermore, as a semi-crystalline material, 100% crystallinity will never be reached, commercially available semi-crystalline polymers with 60% crystallinity are said to be highly crystalline [45]. Throughout this work the terms amorphous and crystalline are used to describe the less crystalline as-sprayed PES and the more crystalline thermally aged PES.

**Table 2:** Reduced moduli from nano indentation

Condition	E$_\mathrm{{r, PES}}$	E$_\mathrm{{r, AlSi}}$
	[GPa]	[GPa]
As-sprayed	5.1 ± 1.3	77.8 ± 8.9
Aged ($200^\circ$C)	8.2 ± 2.1	77.9 ± 10.9

**Fig. 4:**
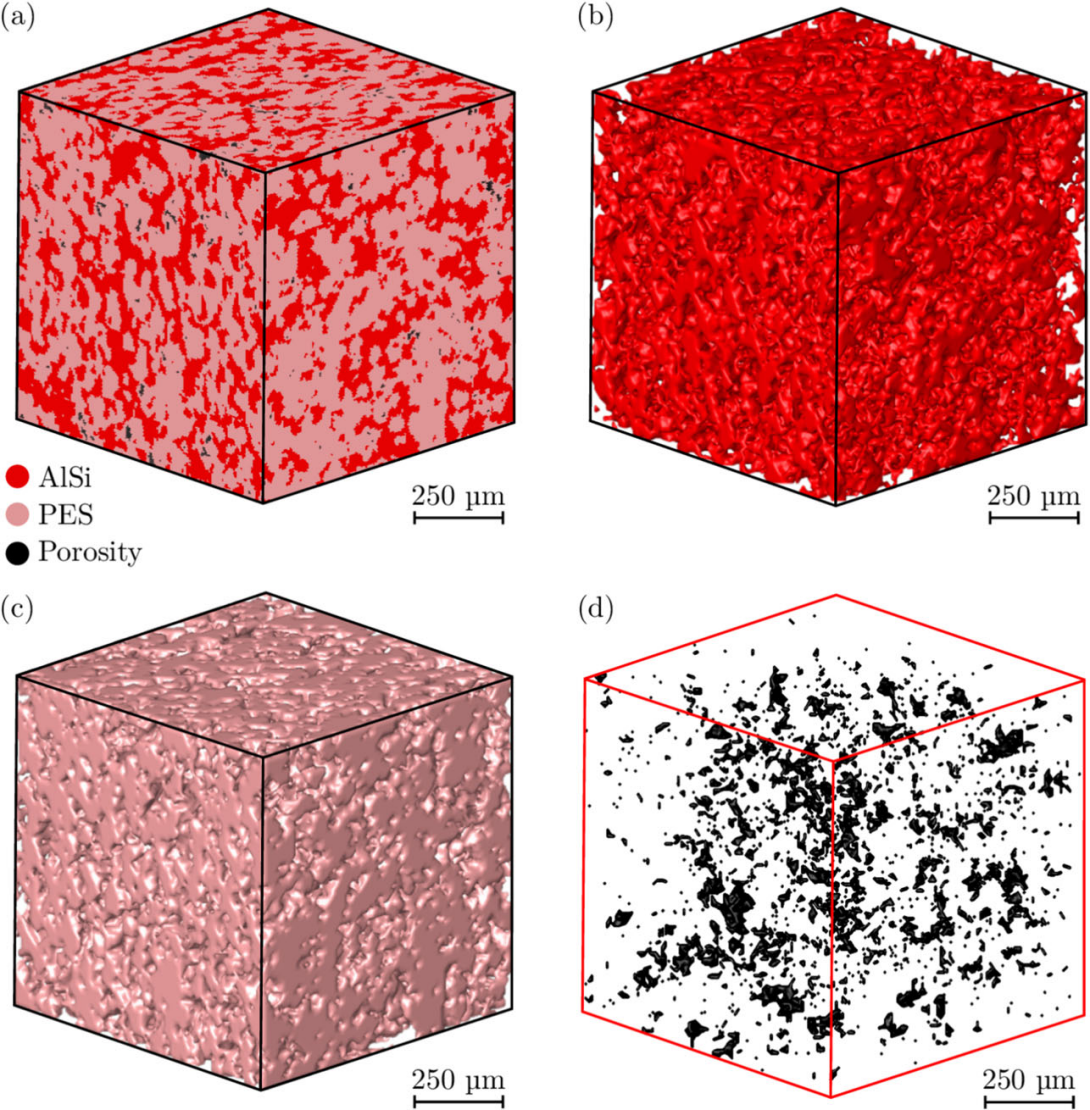
RVE extracted from X-ray CT scan of a compacted sample, with 1 mm edge length. (a) RVE, (b) AlSi phase, (c) PES phase, (d) Porosities

Furthermore, by compacting the samples and causing plastic deformation, the height of the sample heights were slightly reduced whilst their diameters increased. The compacted samples had mean height of 3.35 mm, with a standard deviation of 0.06 mm, while the mean compacted sample diameters was 6.2 mm. An XCT scan of a single compacted sample obtained using the same parameters for the as-sprayed specimen is shown in Fig. 4. The overall structure remained similar to that seen for the as-sprayed sample, but with significantly fewer porosities. Here the AlSi, PES, and porosity phase volume fractions were 36.5, 62.6, and 0.9% respectively. When compared with the as-sprayed material, the compacted abradable showed a significant decrease of 82% in the porosity volume fraction.

### Quasi-static Tests

Quasi-static compression tests were carried out at a strain rate of 0.01 s$^{-1}$, using all four specimen conditions at room temperature, $110^\circ$C, and $200^\circ$C. These temperatures capture material phase changes within the normal operating range, and will provide an insight in to the differing failure behaviours between conditions, and in conjunction with the high-rate tests, the rate dependence. The specimens were placed between a steel anvil and die with parallel edges, and heated via an induction coil. Once the target temperature was reached, as measured by a thermocouple mounted on the surface of the anvil near the specimen, a 15 min hold period was implemented before beginning the test to ensure the specimens were at a uniform temperature. The tests were ran until a total specimen strain of 25%, as measured by a contact extensometer placed on the anvil and die, just below the and above the specimen respectively.

### High-rate Tests

**Fig. 5:**
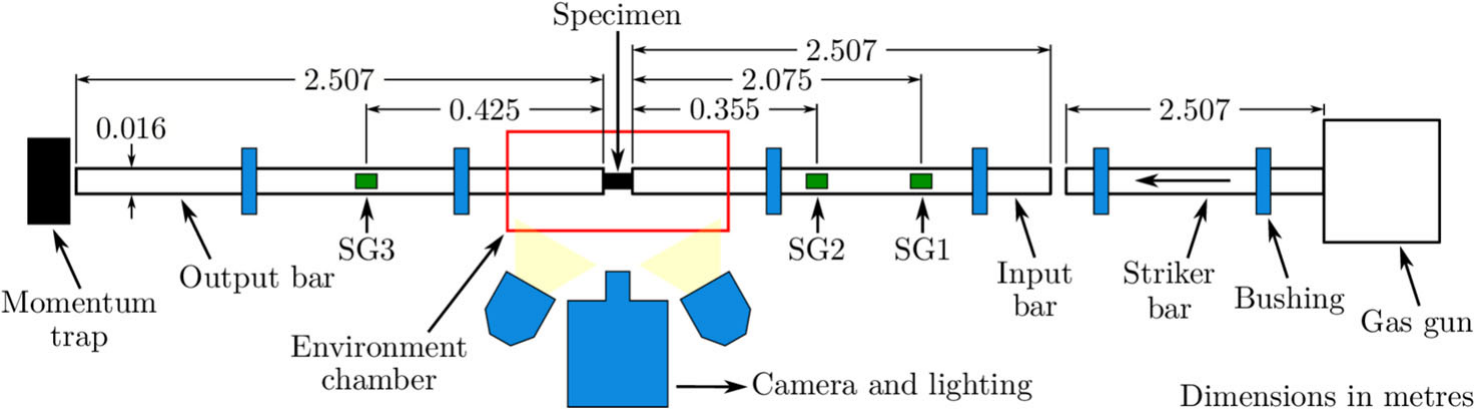
Schematic representation of the SHPB apparatus

The high-rate compression tests were conducted on the SHPB apparatus as shown in Fig. 5, with strain rates in the region of 650 - 850 s$^{-1}$. The SHPB apparatus comprised a striker bar which was fired via a compressed air gun into the input bar, causing a stress wave to travel through the bars and specimen. As the stress wave reached the end of the input bar, it was partially transmitted through the sample to the output bar, and partially reflected back through the input bar. All of the bars were supported by low-friction nylon bushings to prevent any lateral bar movements, and to ensure that the bars remain aligned. An environment chamber was used to heat the specimens for a duration of 15 min before testing, this ensured a uniform temperature through the sample, and finally, high speed video footage was recorded using a Kirana 1 M camera, with a frame rate of 300,000 fps.

The bars were assumed to be thin, and as such a one-dimensional analysis procedure, based on the method of characteristics and D’Alambert’s solution of wave equations, where the volumetric and rotational strains are negligible, was used to calculate the longitudinal forwards and backwards travelling strain wave magnitudes. In this case, the stresses, $\sigma$, and particle velocities, *v*, at any given position and time are

2 $$\begin{aligned} \sigma (x,t)= &   \alpha (x,t)+\beta (x,t)\text {, and} \end{aligned}$$

3 $$\begin{aligned} v(x,t)= &   \frac{1}{\rho c}[\beta (x,t) - \alpha (x,t)]\text {.} \end{aligned}$$

Here $\rho$ is the density of the bars, *c* is the speed of sound within the bars, while $\alpha (x,t)$ and $\beta (x,t)$ the forward and backward travelling stress waves as a function of position, *x*, and time, *t*.

As precise short strain gauges were attached to the surfaces of the input and output bars, the longitudinal stress amplitudes and their velocities in the bar cross sections were calculated directly from the measured strains using

4 $$\begin{aligned} \sigma (x,t)= &   E\varepsilon (x,t)\text {, and} \end{aligned}$$

5 $$\begin{aligned} v(x,t)= &   \frac{\sigma (x,t)}{\rho c}\text {,} \end{aligned}$$

with *E* being the Young’s modulus of the bars, and $\varepsilon$ the measured strains.

The SHPB used in this study was specifically designed for dynamic compression testing of materials with low stress wave propagation velocities, which require long-duration stress pulses to achieve dynamic equilibrium, particularly under large deformation strains. To accommodate these conditions at the proposed strain rates a 2.507 m striker bar, comparable to the incident and output bar lengths, was used to generate stress pulses exceeding 1 ms in duration. While this resulted in the superposition of incident and reflected waves at the gauge location, it was necessary to ensure adequate pulse duration and accurate measurements. The duration of the resulting stress pulse, $t_{pulse}$, was described by

6 $$\begin{aligned} t_{pulse} = \frac{2l_{striker}}{c_{striker}}\text {,} \end{aligned}$$

with $l_{striker}$ and $c_{striker}$ being the length of and speed of sound within the striker bar respectively. The algorithm, as described by De Cola et al. [46] and Zhang et al. [47], was used to separate these waves in order to determine the particle stresses and velocities at the interfaces in contact with the specimen by using the measurements obtained from the strain gauges.

The interface surface stresses and velocities were then used to determine the engineering stress and strain of the specimen at any given time. With the average force between the bar ends that was used to calculate the mean engineering stress in the specimen given by

7 $$\begin{aligned} \begin{aligned} F(t) =&\frac{1}{2}\{A_{inp}[\alpha (x_{max}^{inp},t) + \beta (x_{max}^{inp},t)] \\&+ A_{out}[\alpha (x_{min}^{out},t) + \beta (x_{min}^{out},t)]\}\text {,} \end{aligned} \end{aligned}$$

where *A* was the cross sectional area of the bars. The results were deemed to be valid if and when dynamic equilibrium was achieved; that is, when the stresses at the specimen interfaces with the input and output bars are approximately equal [48, 49]. The achievement of force equilibrium conditions ensures that deformations are approximately uniform along the gauge length of the specimen. These conditions are generally accompanied by the achievement of approximately constant strain rate during the experiment.

**Table 3:** Test parameters used for the SHPB test campaign

Sample conditioning	Temperature	Strain rate	Diameter	Height
	[$^{\circ }$C]	[s$^{-1}$]	[mm]	[mm]
As Sprayed	RT, 110, 200	650 - 850	6	3.5
Aged	RT, 110, 200	650 - 850	6	3.5
Compacted	RT, 110, 200	650 - 850	6.2	3.35
Aged & Compacted	RT, 110, 200	650 - 850	6.2	3.35

The aspect ratio, *l*/*d*, with *l* and *d* being the specimen length and diameter respectively, was also of significant importance, with previous studies suggesting a ratio between 0.5 and 2 to avoid buckling in homogenous materials. However, in this work a heterogeneous abradable was used, and therefore shorter specimens were more desirable to increase the strain rate, strain distribution through the specimen, and to improve the dynamic equilibrium conditions [46, 50]. Furthermore, uncertainties can arise with inhomogeneous specimens that are too small. As previously discussed, a conservative RVE has a volume of 1 mm$^{3}$. Therefore, the specimens used in this work had a suitably small aspect ratio, while being large enough to accurately represent the material. The test parameters used for the high strain rate tests are shown in Table 3.

The proposed strain rates enured that force equilibrium was achievable, while the temperatures were within the normal operating range of the abradable and capture key phase changes, particularly in the PES phase. It is worth noting that a small portion of the bars are also heated within the environment chamber which can lead to local changes in the elastic modulus of the bars. The discontinuity in elastic modulus of the bars within the environment chamber can alter the stress wave propagation velocity and distort elastic waves. Furthermore, this may also lead to inaccuracies regarding strain measurements as the heat is conducted through the bars. However, given the elastic modulus of the Ti-6Al-4V bars is relatively constant within this temperature range and the limited length of the bars within the environment chamber, these thermal effects are deemed to be negligible [51–54]. The variation in Young’s modulus and density between room temperature and $200^\circ$C corresponds to a change in stress wave propagation velocity of 4.38%, which can be considered negligible given the short length of the affected sections within the environmental chamber [55]. Moreover, this conclusion is further corroborated by the attainment of dynamic equilibrium, assessed using the algorithm described by De Cola et al. [46] and Zhang et al. [47], which employed strain gauge readings from multiple locations on both the incident and transmitted bars.

**Fig. 6:**
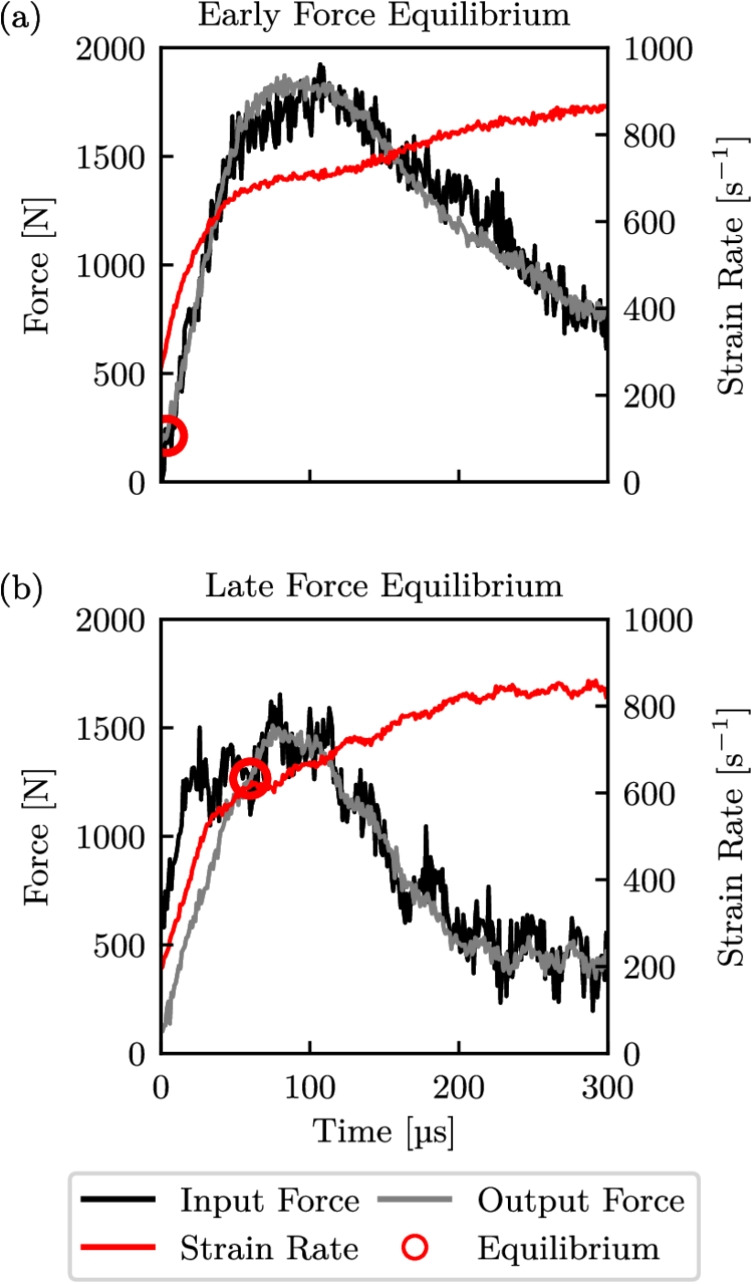
Test forces and strain rates highlighting early and late force equilibrium attainment

### Test Data Analysis

To verify the validity of the high-rate experiments, each test was checked for dynamic force equilibrium and a constant strain rate, as shown by the time histories of the forces and strain rate in Fig. 6. Here the black and grey lines correspond to the input and output bar forces respectively, the red line is the strain rate, and the red circles highlight where force equilibrium was first deemed to occur. In order to determine the onset of force equilibrium the signal data was split into bins of 50 points, and equilibrium was chosen to be at the location where the root mean squared error (RMSE) first fell below 150 N, which was approximately 10% of the mean maximum force. Failure was defined as the point at which the abradable material lost its load-carrying capability, corresponding to catastrophic breakdown due to extensive particle debonding. This point was identified analytically by examining the stress–strain response, specifically by calculating the gradient over bins of 50 data points. Failure was deemed to have occurred when the gradient became negative in five consecutive bins, with the first point in this sequence taken as the failure point. Given the data acquisition rate of 25 MHz, this method provided a temporal resolution of 2 µs. All stress–strain curves in this work have been clipped at this failure point. In the case of an AlSi-PES abradable, failure was catastrophic with the material losing structural cohesion and load-bearing ability, which in this context represents functional failure. Fig. 6(a) is an example of force equilibrium being readily achieved almost immediately, with a constant strain rate throughout the duration of the test prior to failure. In contrast, the force equilibrium onset for the test shown in Fig. 6(b) was one of the latest, and there was a slightly more pronounced rising strain rate prior to failure. However, this test was still deemed to be valid as force equilibrium occurred before the specimen failed, and the increase in strain rate before failure was small and within the predefined test bounds. This also verified that the adopted pulse shaping technique of using a light application of high vacuum grease and 80 gsm paper was appropriate for removing high frequency components from the input pulse and creating an adequately consistent strain rate [56, 57]. While a small amount of noise was still present in the input force signals, this was primarily due to the high sampling rate used to capture the stress waves, while necessary to preserve temporal resolution, can introduce minor oscillations. Additional noise arises from the method used to calculate the incident force, which involves summing the measured incident and reflected waves after applying a time shift to synchronize them at the specimen–bar interface. This process can introduce small fluctuations due to the superposition of two stress waveforms with slightly different amplitudes and timing.

**Fig. 7:**
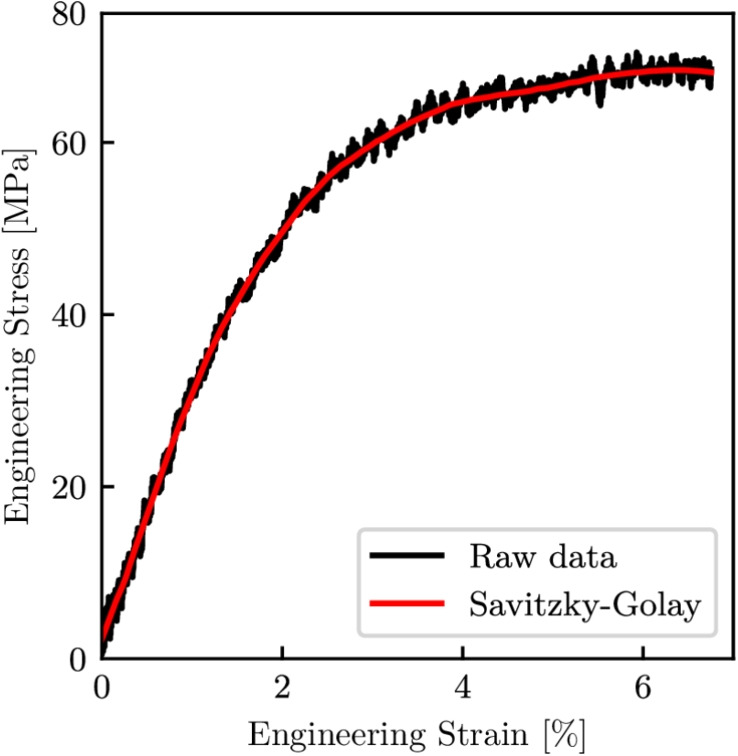
Data before and after applying a Savitzky-Golay filter

When processing the data, engineering stresses and strains were smoothed through the use of a third order Savitzky-Golay filter, as shown in Fig. 7, with the black and red lines corresponding to the raw and filtered data respectively. The filtering process was implemented to make it easier to identify when the specimens began to fail as the gradient turns negative, in addition to removing the noise which aided in the determination of failure stresses and strains.

To obtain parametrised stress strain curves, the Ramberg-Osgood (RO) relationship using Holloman parameters as described by

8 $$\begin{aligned} \varepsilon = \frac{\sigma }{E}+\left( \frac{\sigma }{K} \right) ^{\frac{1}{n}}\text {,} \end{aligned}$$

with $\varepsilon$ being the strain, $\sigma$ the stress, *E* the Young’s modulus, *K* the strength coefficient, and *n* a unitless strain hardening coefficient [58, 59]. The RO curves were fitted to the filtered test data using the non-linear least squares method, by adjusting *E*, *K*, and *n*, until the convergence criteria of no changes greater than 0.1% with a patience of 50 was met.

### Blade Rub Model

**Fig. 8:**
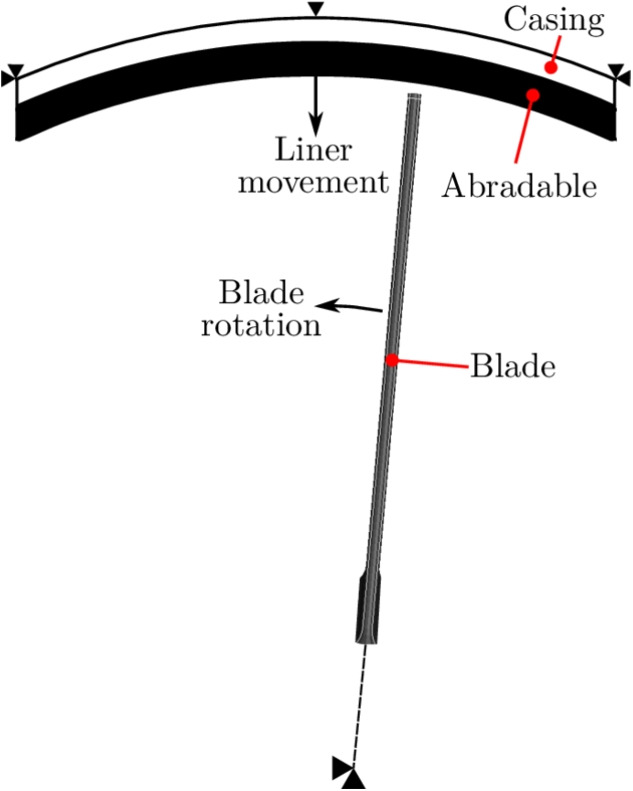
Schematic representation of blade casing interaction model

To investigate the effects of changing abradable properties on the blade response during blade-casing interaction a model was created using LS-Dyna. The simulations used a rotational blade speed of 11,500 RPM (320 m$\cdot$s$^{-1}$) and lasted for a total of six rubs with an incursion rate of 5 µm$\cdot$pass$^{-1}$, followed by two full revolutions without any blade-casing contact so that the blade response could be examined. This limited number of rubs was adequate as full-chord contact could be guaranteed from the onset and a stable behaviour was observed. A schematic of the model is shown in Fig. 8, with the abradable material being attached to a steel casing segment, and a generic straight blade with a tip chord length and thickness of 30 mm and 2 mm respectively, as used by Wollmann et al. [16].

**Fig. 9:**
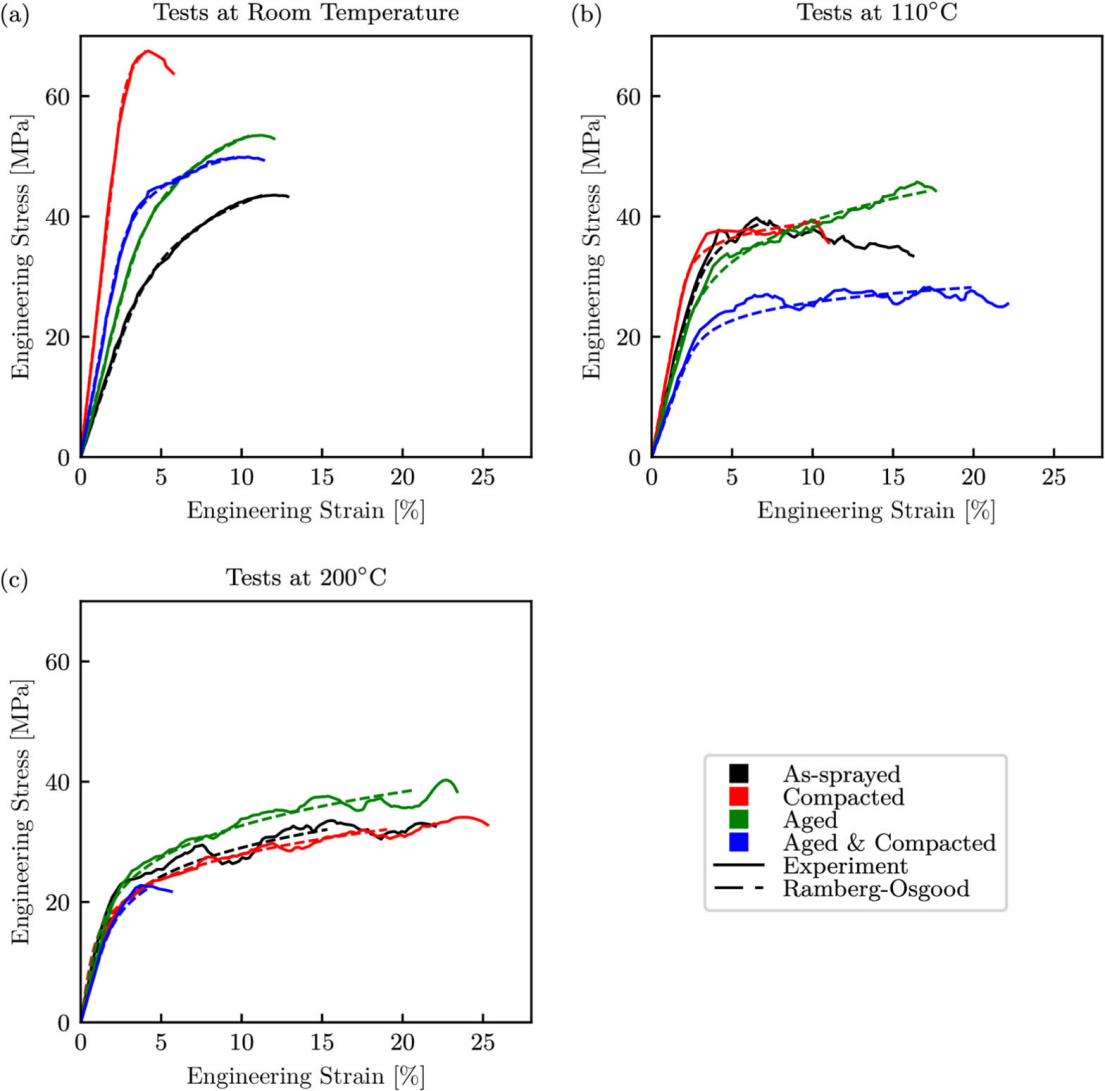
Stress–strain curves for the quasi-static tests. (a) Room temperature, (b) $110^\circ$C, (c) $200^\circ$C

The top surface of the casing was only allowed to move vertically, so that it could advance towards to the blade. The AlSi-PES abradable used had varying properties, corresponding to those obtained from the experimental test campaign in the work. The titanium (Ti-6Al-4V) blade was considered to be elastic with a Young’s modulus of 113 GPa, with the bottom surface of the blade constrained so that it could rotate about the axial axis only. For the contact, a static coefficient of 0.47 and dynamic coefficient of 0.05 were used [40]. The frictional contact limit is described by

9 $$\begin{aligned} F_{lim} = VC \times A_{cont}\text {,} \end{aligned}$$

where *VC* is the coefficient of viscous friction, and $A_{cont}$ the area of the segment in contact with a penetrating node. *VC* was then equated to the yield stress in shear,

10 $$\begin{aligned} VC = \frac{\sigma _y}{\sqrt{3}}\text {.} \end{aligned}$$

The parametrised data from the high-rate experimental testing was then used to define the model material inputs, where the proof stress at 0.2% plastic strain was taken to be $\sigma _y$.

## Results

The results of the quasi-static and high-rate compression tests are presented in the following section. Ramberg-Osgood curves were fitted to the individual quasi-static curves, while the mean fit is given for the repeated high-rate tests. These parameterised curves provide useful inputs for modelling different strain rates and abradable conditions.

**Table 4:** Ramberg-Osgood parameters determined from the quasi-static compression tests

Condition	Temperature	E	K	$\frac{1}{\text{ n }}$	RMSE
	[$^{\circ }$C]	[MPa]	[MPa]	[-]	[MPa]
As-sprayed	RT, 110, 200	794, 1060, 1267	67, 55, 48	6.5, 10, 5.1	0.5, 1.5, 1.8
Compacted	RT, 110, 200	2341, 1454, 1750	83, 46, 45	21.0, 15.8, 5.2	1.5, 1.1, 0.8
Aged	RT, 110, 200	1046, 1010, 1100	77, 62, 54	7.8, 6, 5.1	0.5, 1.4, 1.4
Aged & compacted	RT, 110, 200	1437, 737, 936	64, 34, 44	10.9, 9.8, 5.9	0.4, 1.6, 0.6

### Quasi-static Tests

The stress–strain responses for all of the quasi-static tests, grouped by test temperature, are shown in Fig. 9. Here the solid black, red, green, and blue lines corresponding to samples in the as-sprayed condition, compacted, thermally aged, and both compacted and thermally aged respectively, whilst the dashed lines of the same colours are the Ramberg-Osgood fits. The peak compressive stresses for all of the specimen conditions appeared to follow the same general trend, with the maximum stresses seen at room temperature, which then decreased as the temperature was increased. However, the failure strains and stiffnesses did not follow a common trend for all specimen conditions. The as-sprayed specimens experienced an increase in failure strain and a relatively consistent Young’s modulus with temperature. At room temperature, the pre-compacted specimens behaved in a brittle manner, whilst at $110^\circ$C, comparing with the as-sprayed specimen, a reduction in failure strain equal to the pre-compaction strain was seen. The significant increase in the peak compressive stress at room temperature, is an indication that at lower temperatures the removal of porosities has a significant influence on the Young’s modulus and yield of the material. The thermally aged specimens showed an increase in peak compressive stress at all temperatures in comparison to the baseline as-sprayed condition, and also had a greater strain hardening rate at $110^\circ$C. This suggests that the increased crystallinity of the polyester phase increased the overall breaking strength of the abradable and improved its thermal behaviour. Finally, at room temperature the tests conducted with aged and compacted specimens, behave in a similar way to the equivalent test with aged specimens, while the tests at elevated temperatures saw a significant reduction in load carrying capability.

**Fig. 10:**
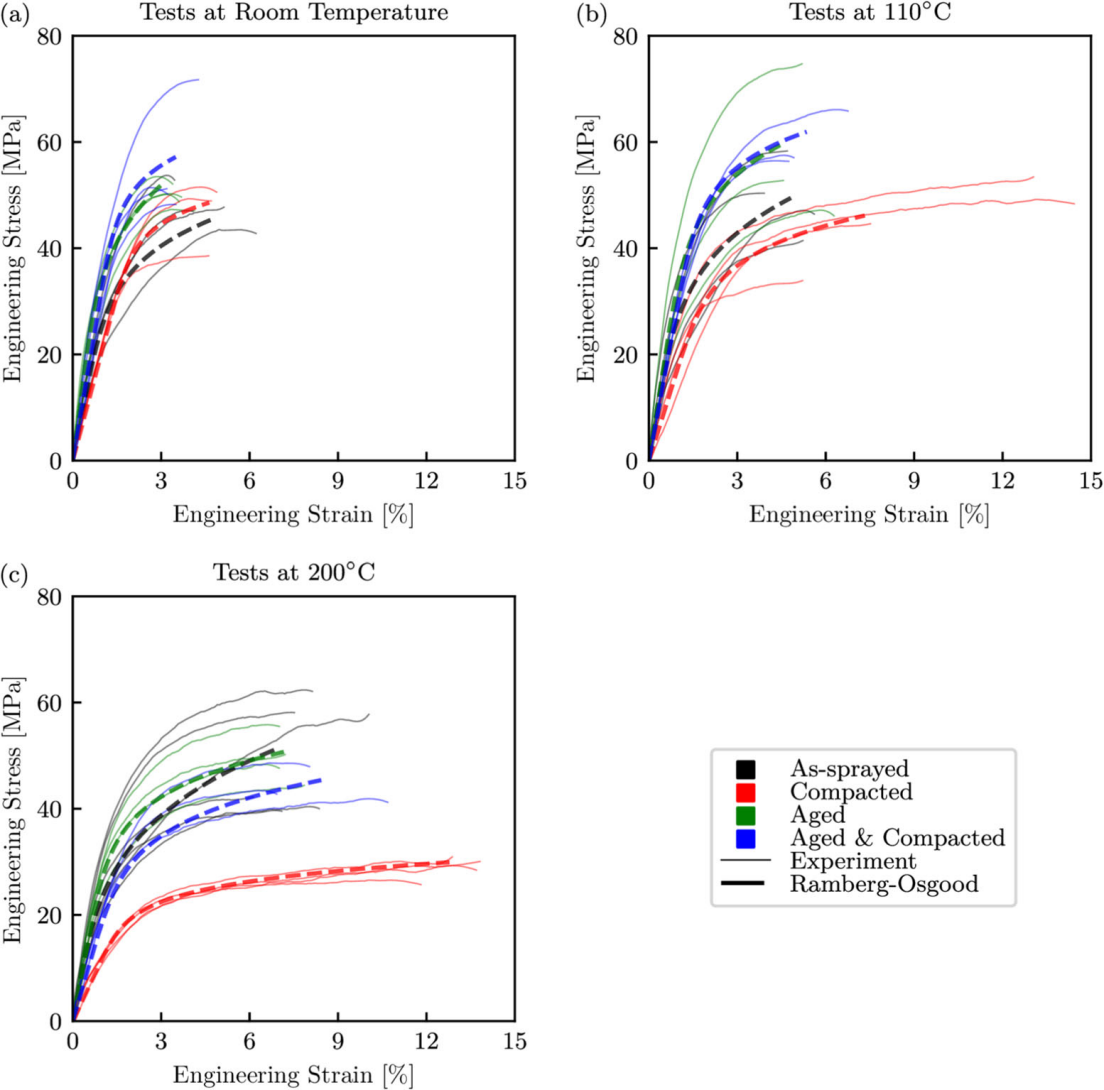
Stress–strain curves for the high strain rate tests. (a) Room temperature, (b) $110^\circ$C, (c) $200^\circ$C

Ramberg-Osgood curves were fitted to the experimental results in order to provide parametrised material models, and were in good agreement with the experimentally obtained data. The necessary parameters to recreate these curves are presented in Table 4, with the maximum root mean squared error (RMSE) being 1.8 MPa. The observed increases in stiffnesses, which were particularly apparent for the as-sprayed specimens, can be attributed to the random distribution of constituent phases within the material, in addition to an increase in adhesiveness of the PES with the anvils at high temperature. It is interesting to note that this increase in Young’s modulus was less pronounced for the aged specimens where the PES had greater thermal stability.

**Table 5:** Ramberg-Osgood parameters determined from the high strain rate SPHB tests

Condition	Temperature	E	K	$\frac{1}{\text{ n }}$
	[$^{\circ }$C]	[MPa]	[MPa]	[-]
As-sprayed	RT, 110, 200	2800, 3370, 3040	82, 103, 108	5.9, 4.6, 4.0
Compacted	RT, 110, 200	2100, 1790, 1190	68, 71, 40	11.2, 7.0, 7.7
Aged	RT, 110, 200	4280, 3620, 2880	113, 103, 77	5.2, 6.5, 6.9
Aged & compacted	RT, 110, 200	3310, 3010, 1910	85, 90, 71	10.3, 9.1, 6.2

### High-rate Tests

Using a similar approach to the quasi-static stress–strain curves, those obtained from the high-rate testing are presented in Fig. 10. The bold lines correspond to the mean Ramberg-Osgood fits of the experimental data of the same colour, with the parameters shown in Table 5. It is important to note that while Young’s modulus is a parameter of the Ramberg-Osgood relationship, it is often impractical to determine these properties from SHPB tests, and therefore should only taken as indicative values [60]. For the specimens that had been either compacted, or aged and compacted, the general trend is the same as that seen for the quasi-static tests, with the maximum stresses decreasing and failure strains increasing with temperature. With the removal of porosities in these cases, the loss of load carrying ability at $200^\circ$C can be attributed to the softening of the polyester phase, with the compacted specimens seeing a greater loss as the polyester in this case comprised a greater amount of amorphous regions. In contrast, distinguishing between different temperatures was more challenging for the as-sprayed and aged specimens. However, the failure strains for the high-rate tests follow the same trend as those seen under quasi-static conditions, with an increase in temperature resulting in a more ductile response.

## Discussion

In this section comparisons between each of the abradable conditions are made over all test temperatures and for both the quasi-static and high-rate loading. T-tests were used to determine where statistical differences between the tests existed, while Eshelby’s inclusion theorem was used to highlight the differences in stress distribution depending on the inclusion Young’s modulus and to infer how this influences the failure behaviours. Finally, blade-casing interaction models are presented to highlight the influence of abradable constitutive properties on the blade response.

### Quasi-static Response

**Fig. 11:**
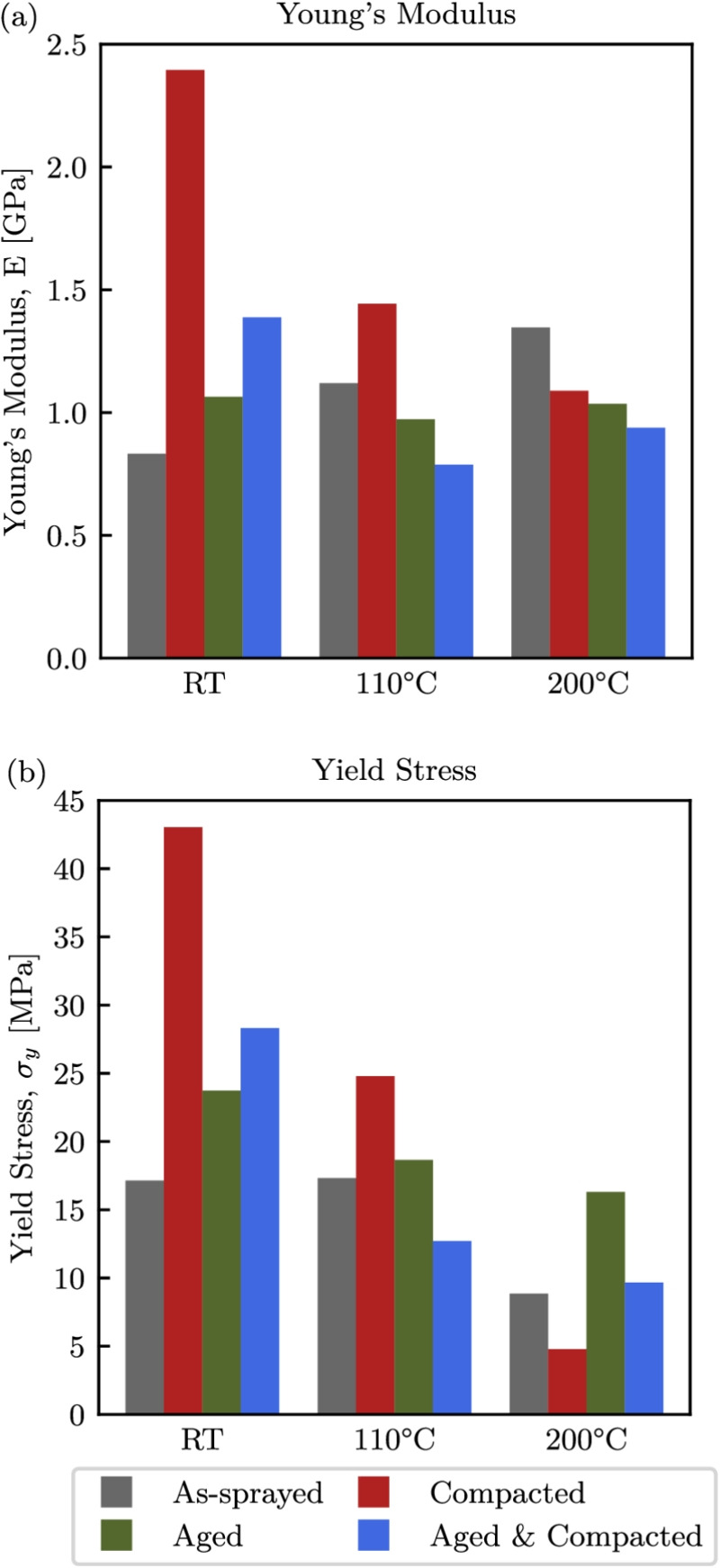
Young’s moduli (a), and yield stresses (b), for all specimen conditions under static loading

Before examining high-rate tests, it is useful to first consider the quasi-static response, as accurate stiffnesses and yield stresses can only be reliably extracted from these tests due to the non-uniform stress distributions typical in SHPB setups [60]. Therefore, the obtained stiffnesses and yield stresses are presented in Fig. 11 for the quasi-static tests only. From Fig. 11(a), it can be seen that the Young’s modulus of the as-sprayed material increased with temperature, while that of the aged specimens was relatively consistent over all temperatures with a value in the order of 1 GPa. The compacted specimens have a much greater Young’s modulus of 2.4 GPa at room temperature. The increasing Young’s modulus of the as-sprayed specimens can be attributed to increasing friction with temperature at the anvil interface as the PES phase softened preventing the material at the interfaces from moving radially outward, this phenomena was not seen for the more thermally stable aged specimens. For the specimens that were pre-compacted, a drop in Young’s modulus was seen with increasing temperature, which suggests that the AlSi phase, which has a better thermal stability than the polyester, carries a large part of the initial load, and upon compaction, thin struts of AlSi fail [26]. This means that the stress is more uniformly distributed through both phases, and becomes heavily dependent on the state of the polyester as it transitions from glassy to rubbery. When looking at the yield stresses in Fig. 11(b), the as-sprayed specimens saw a reduction in yield strength at $200^\circ$C as both phases softened. The loss of yield strength is much more drastic for the compacted specimens, again because of the loss of load carrying capabilities in the AlSi. It can also be seen that thermal ageing had a significant effect, which is most clearly seen at $200^\circ$C, with both aged specimen types exhibited increased yield strength in comparison to their as-sprayed counterparts. The thermally aged specimens have a greater degree of crystallinity compared to those that have not been aged. Therefore, as there were more crystalline regions, which do not experience a glass transition and subsequent bulk change to a rubbery state, the polyester phase had greater thermal stability [61]. From Fig. 3, it can be seen that for these crystalline regions, melting will begin to occur at $320^\circ$C, which is on the operational temperature limit of the abradable [4].

### Identification of Statistical Differences Between Specimen Conditions during High-rate Testing

**Fig. 12:**
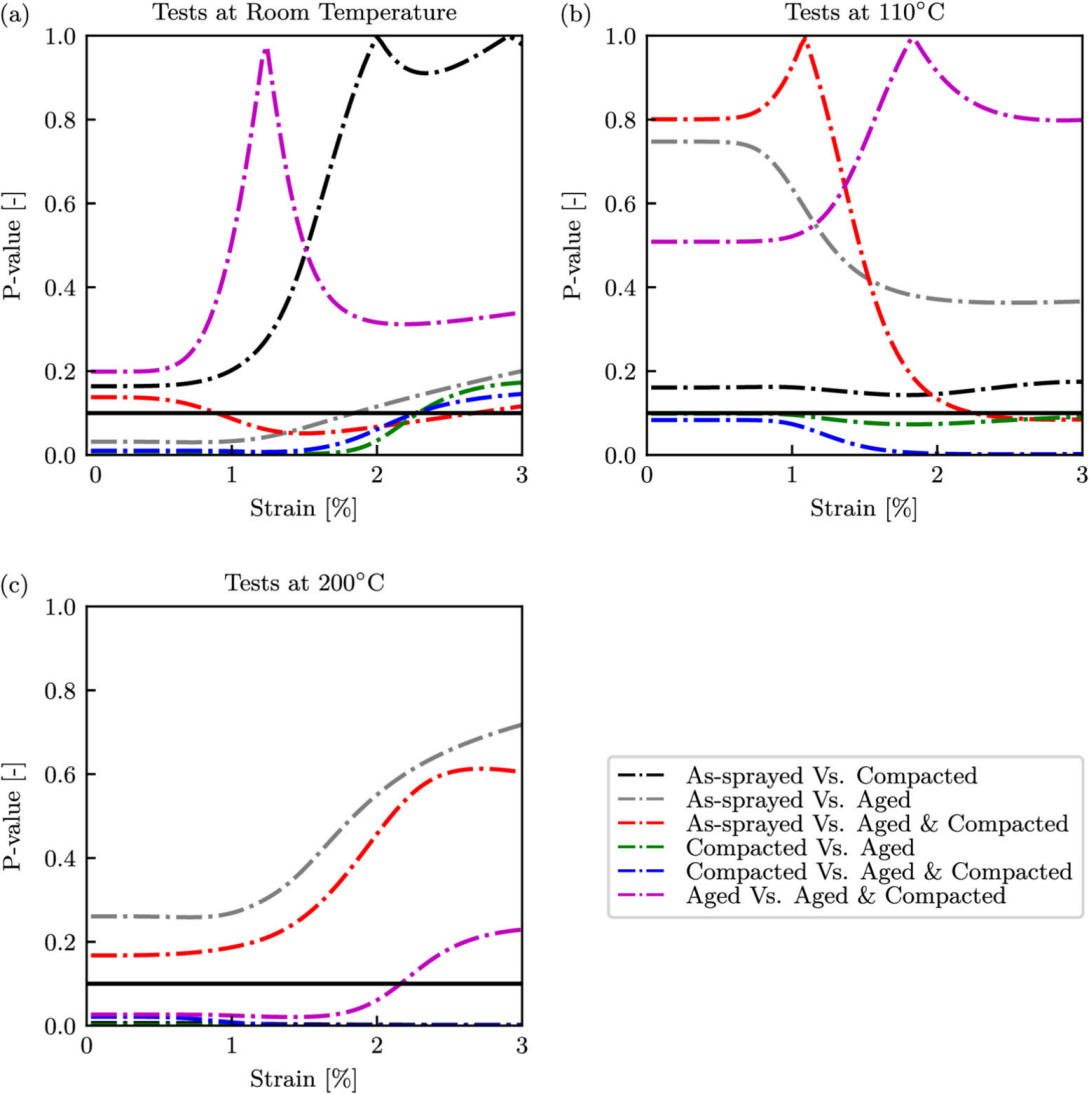
P-values from t-tests comparing all specimen conditions at like temperatures for the dynamic tests. (a) Room temperature, (b) $110^\circ$C, (c) $200^\circ$C

To help identify statistical differences that exist between all of the pre-conditions at a given temperature, a series of two tailed t-tests were conducted using an alpha value of 10%, as this reduces the amount of evidence needed to reject the null hypothesis. These t-tests compared the stress values at like strains of the differing pre-treatment types up to a maximum strain of 3%, as this was the earliest failure seen amongst all tests and enabled a complete comparison. The p-values obtained are presented in Fig. 12, with the horizontal black lines representing alpha value. At room temperature, there is no difference between the specimens that had been aged and those that were both aged and compacted, or between as-sprayed and compacted specimens. All of the other specimen pairings showed a statistically significant difference up to 1.5% strain before these differences subside by 3% strain. Of these groups, they all contained one specimen that had been aged and one that had not, implying that thermally ageing had an influence on the response of the samples. For the tests conducted at $110^\circ$C, initially only those groups comparing compacted specimens with those that had been aged or aged and compacted were different from each other. While at 3% strain the as-sprayed specimens behave differently to those that had been aged and compacted. Finally, at $200^\circ$C statistically significant difference were observed when comparing the compacted specimens with those that were as-sprayed or aged. It appears that both ageing and compacting the specimens had a statistically significant influence on the behaviours of the abradable when compared to an as-sprayed sample. Compacting removed the porosities within the abradable, removing voids for particles to relocate as the material deformed, while also introducing cracks. The specimens that had not been compacted appeared to show greater variation, likely a consequence of the random porosity distribution. Ageing increased the crystallinity and hence the Young’s modulus of the PES phase, while also improving its thermal stability.

### Comparison between Quasi-static and High-rate Tests

**Fig. 13:**
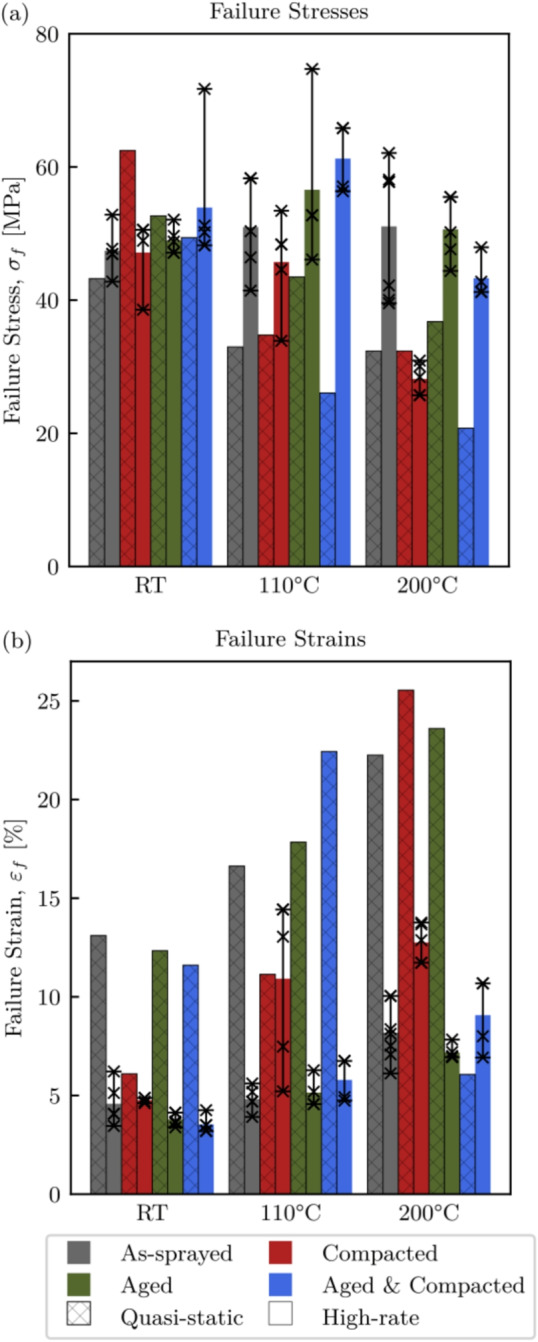
Engineering failure stresses (a), and strains (b), for all specimen conditions under quasi-static and high-rate loading

The failure stresses and strains for the quasi-static and high-rate tests are shown in Fig. 13(a) and (b) respectively. Here the quasi-static test data is hatched while the high-rate data is not. For the high-rate tests, the individual bars represent the mean values for any given test type and temperature, while the error bars and scatter points show the minimum and maximum values obtained, and the distribution respectively. It is clear that this material exhibits a strain rate dependence for all conditions, and it becomes more pronounced with temperature. When considering the failure stresses, there was little change with temperature for the high-rate tests, with the compacted specimens being an exception. Conversely, the quasi-static tests experience a significant drop in failure stress with temperature. As for the failure strains, there is generally an increase for the quasi-static tests, with only the aged and compacted specimen showing a significant decrease at $200^\circ$C. To a lesser degree, an increase in failure strain is also seen for the high-rate tests, with the most significant increase being with the compacted specimens and the other specimen types demonstrating a similar behaviour. However, at the highest temperature the aged specimens can be seen to fail at a slightly lower strain than those in the as-sprayed condition, while the aged and compacted specimens had a significantly lower failure strain when compared to those that were compacted only.

### Effects of Strain Rate and PES Condition

Strain rate appears to play a significant role in influencing the failure behaviours of abradable materials. The abradable consists of AlSi and PES particles along with porosity, giving it characteristics similar to a granular material. At low strain rates, particles may first undergo elastic deformation at contact points, followed by densification through slippage and gradual realignment. At higher strain rates, however, the response may favour more immediate particle reorientation to accommodate the applied load [46, 62]. This behaviour is consistent with mechanisms reported in the literature for granular systems and is proposed here as a plausible explanation based on the experimental observations.

The state of the polyester phase may also significantly influence the ease with which particle realignment occurs. After ageing, the polyester contains a greater proportion of crystalline regions, making it more brittle and thermally stable than the as-sprayed polyester. With a more crystalline structure, it is hypothesised that particle reorientation could be hindered, as the particles likely do not deform as readily and thus become more dependent on the strength of bonding with the surrounding matrix. In contrast, when the polyester is more amorphous, particle realignment under high strain rates may be more easily facilitated. In this state, the polymer chains exhibit greater mobility and deformation within the polyester particles themselves can occur more readily [63], thereby reducing the likelihood that failure of the bonding surfaces occurs first. This may allow for increased particle rearrangement and contribute to the overall plasticity and deformation behaviour of the abradable.

### Influence of Porosity

In the case of the compacted specimens, large strains to failure were observed. This is believed to result primarily from the ductility of the amorphous polyester phase and the denser particle packing achieved by removing porosities prior to testing. The presence of porosity appears to reduce the overall structural integrity of the abradable, as thin AlSi struts are more prone to failure [26], and also facilitates particle mobility by providing space into which material can relocate.

When the specimens were both aged and compacted, a different behaviour was observed. As previously discussed, thermal ageing increases the crystallinity of the polyester phase, making it more brittle. As a result, the aged and compacted specimens exhibited a more brittle response compared to those that were only compacted. Interestingly, despite this increased brittleness, the failure stresses for the aged and compacted samples were higher. This may suggest that the bond strength between particles in the aged and compacted condition exceeds the yield strength of the polyester phase in the compacted-only specimens. On the other hand, the failure strains were lower for the aged and compacted specimens, supporting the interpretation that once particle debonding initiates, there is limited plastic accommodation, leading to abrupt failure. In contrast, the compacted-only samples appeared to benefit from improved particle mobility due to the ductile nature of the polyester, with fewer broken bonds and a more gradual failure process.

### Specimen Condition Comparisons

In the case of the compacted specimens, large strains to failure were observed. This was primarily due to the ductility of the amorphous polyester and denser packing of particles with the porosities being removed prior to testing. The existence of porosities not only decreases the abradables overall structural integrity as thin AlSi struts exist [26], but also makes particle mobility easier, as there is ample space for material to relocate. However, when the specimens were aged and compacted, a different behaviour was observed. As previously discussed, the polyester phase of the aged specimens was more brittle, and as result, the aged and compacted specimens exhibit increased brittleness when compared to the compacted only samples. Interestingly, despite this increased brittleness, the failure stresses of the aged and compacted samples were higher, suggesting that the bond strength between particles within the aged and compacted samples is greater than the yield strength of the polyester phase in the compacted only samples. However, the failure strains were lower for the aged and compacted, further suggesting that as the particles de-bond they easily relocate and the abradable fails abruptly, whilst the compacted only samples had good particle mobility due to the ductile polyester while the bonds between particles are not broken.

**Fig. 14:**
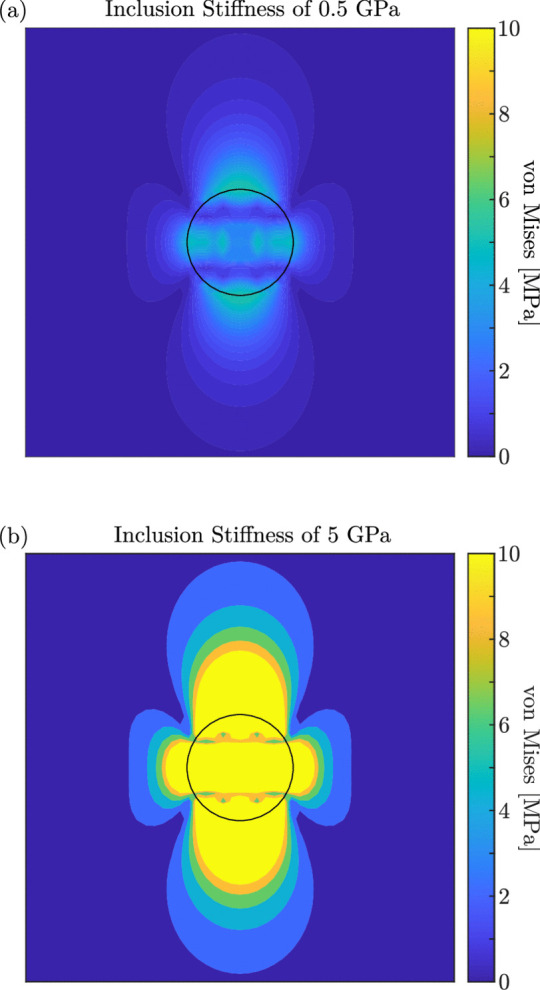
von Mises stress fields obtained using Eshelby’s inclusion theory

The influence of the polyester state on the failure behaviour of the abradable can be demonstrated by using Eshelby’s inclusion theory [64, 65]. This analysis is not intended to replicate specific experimental conditions, but rather to provide a conceptual framework for understanding stress distribution effects arising from different polyester states. Here a polyester inclusion with an initial eigenstrain of 5% in the vertical direction is placed inside, and constrained by an infinite AlSi medium. However, being that the inclusion is restricted by the surrounding AlSi medium a complex stress state arises. Two von Mises stress fields obtained using Eshelby’s inclusion theory are shown in Fig. 14(a) and (b), which correspond to a soft and predominantly amorphous inclusion above $T_g$, and a hard, mostly crystalline inclusion above $T_g$ respectively. The central black circle represents the polyester inclusions perimeter. In both of these models the AlSi medium was considered to have a Young’s modulus of 18 GPa [24] and Poisson’s ratio of 0.08 [5], the Poisson’s ratio of the polyester was kept at 0.25, while the high and low inclusion stiffnesses were 5 and 0.5 GPa respectively [61, 66–68]. For the scenario with a low Young’s modulus inclusion, the polyester was easily deformed at stresses lower than that of the particle bond strength, with the majority of the load is carried by the AlSi matrix material. The stresses at the inclusion and medium interface were much less than 9.7 MPa as reported in the material data sheet [4]. This supports the case that for polyester with a large percentage of amorphous regions, the load carrying capability of the abradable is reduced, particularly at temperature, but can be expected to have large strain to failure, as seen in Fig. 13. Here the failure stresses under high-rate conditions, at room temperature and $110^\circ$C, for the as-sprayed and compacted specimens were approximately 5 MPa less than their thermally aged counterparts. Furthermore, the strains to failure for this same group at room temperature saw a mean value approximately 1% less for the aged specimens. With the high Young’s modulus inclusion, such as after thermally ageing the abradable, the stresses at the inclusion–matrix interface exceed the reported bond strength, making interfacial debonding a likely outcome [69]. Therefore, a thermally aged AlSi-polyester abradable can carry greater loads, but with reduced failure strains.

**Fig. 15:**
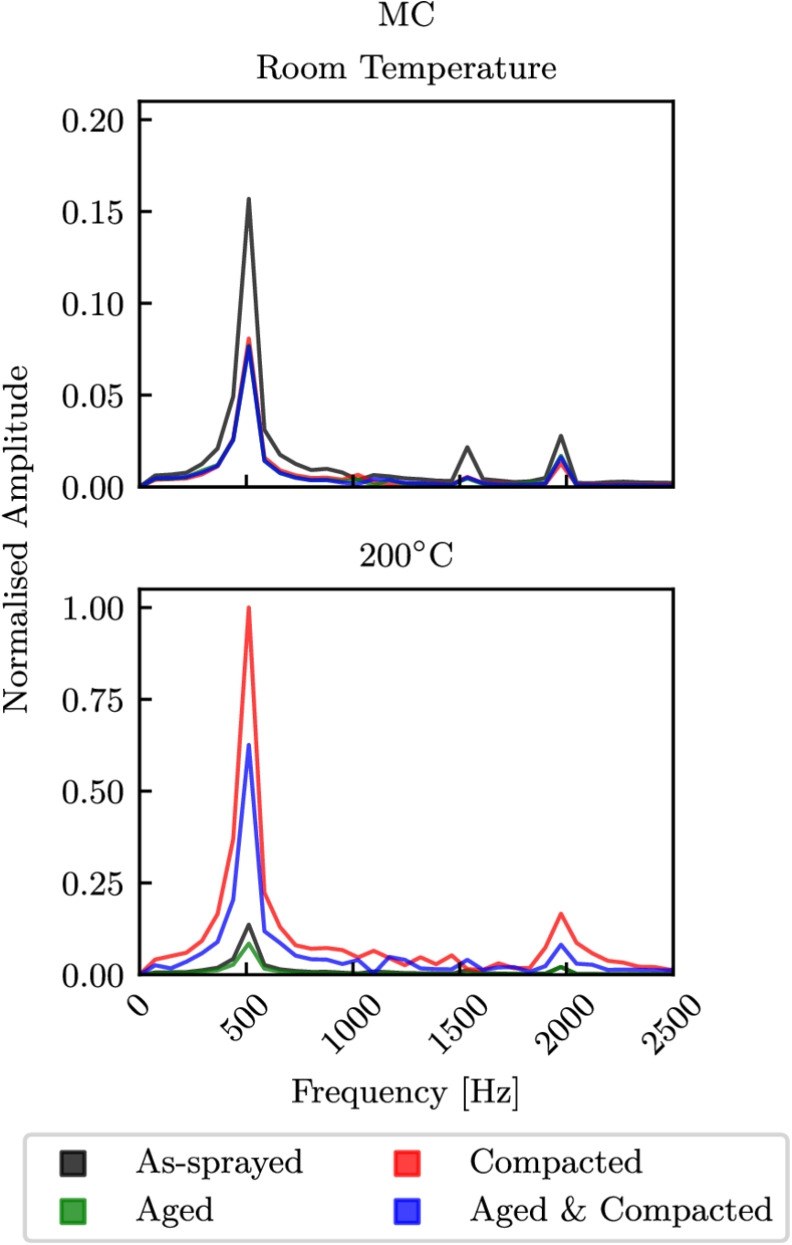
FFTs from virtual strain gauges at the MC position

### Influence of Abradable Properties on Blade Response

**Fig. 16:**
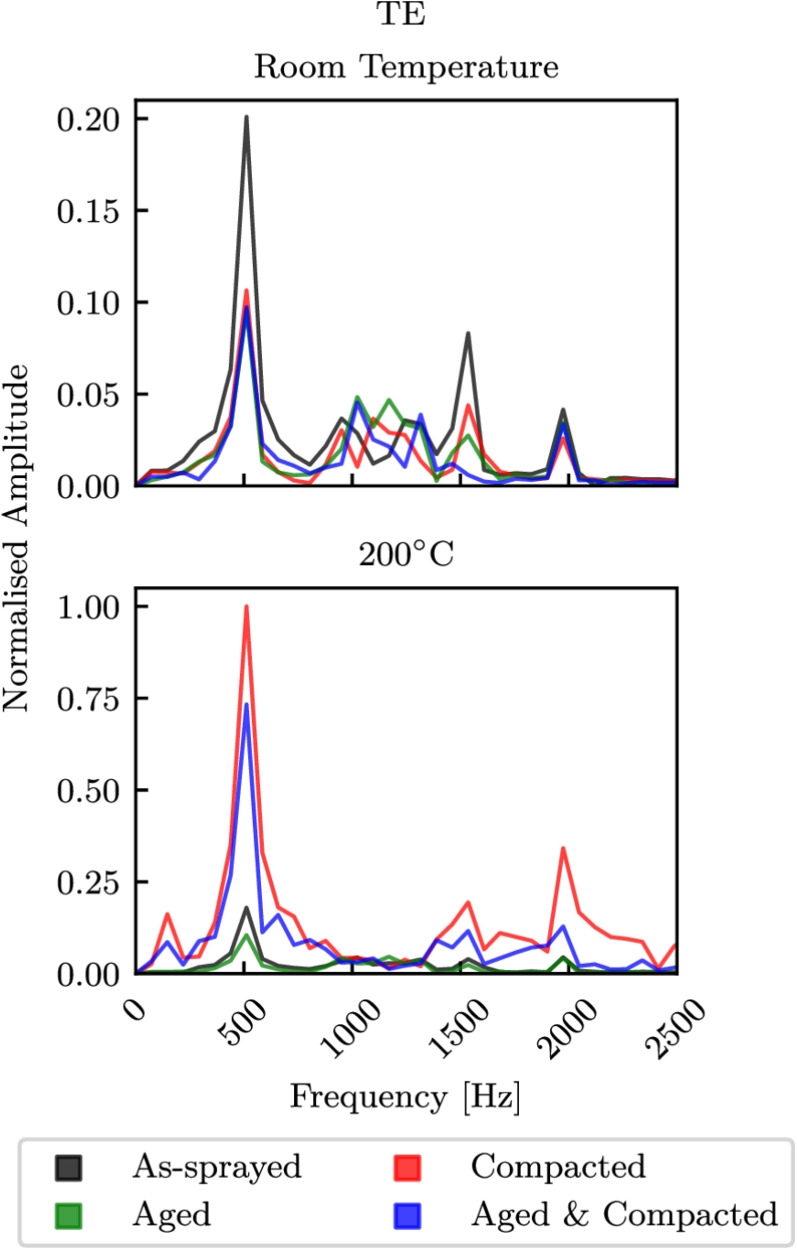
FFTs from virtual strain gauges at the TE position

A series of blade-casing interaction simulations were ran, assuming that the abradable temperature was constant during any given simulation. The material properties used were those obtained from the high-rate compression tests. In reality frictional heating will occur and the strain rates will change depending on incursion rate or blade rotational speed. These simulations provided an insight into the influence of different abradable conditions at an ambient room temperature and at $200^\circ$C on the response of the blade. Fast Fourier Transforms (FFTs) from virtual strain gauges near the blade fillet at the mid-chord (MC) and trailing edge (TE) positions of the model are shown in Figs. 15 and 16 respectively. Here the top plots correspond to models ran with room temperature properties, while those on the bottom correspond to properties obtained from the high-rate tests at $200^\circ$C. Furthermore, the FFTs at either the MC or TE positions have been normalised with respect to the maximum amplitude seen for any condition at the same position, allowing for the change in mode prominence between temperatures for like conditions to be analysed. The three largest peaks from left to right correspond to first flap (1F), first torsion (1T), and second flap (2F). At the MC and TE positions there was little difference between the responses seen with temperature for the as-sprayed conditions, and similarly there was little difference for the aged abradable. However, for the as-sprayed material the torsion peak was more pronounced at room temperature. For the compacted abradable model much more prominent vibrations were seen at $200^\circ$C, with significant increases in 1F and 2F at the MC position, and for all three peaks the TE position. The models with the aged and compacted abradable had a similar response, albeit with slightly less prominent 1T and 2F peaks with respect to 1F.

**Fig. 17:**
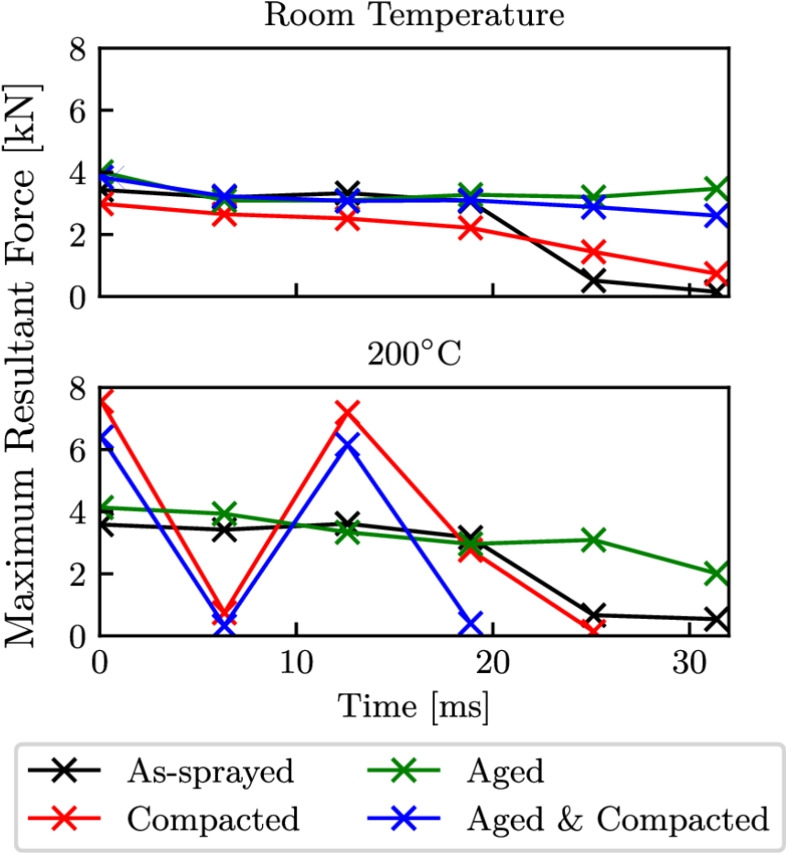
Maximum resultant contact forces seen during each rub of the model

The maximum contact forces that was seen during each rub is shown in Fig. 17, and explains the change in vibration behaviour with temperatures for both the compacted, and the aged and compacted abradable models. These two conditions saw much larger contact forces for the first and third rub, and is attributed to an increase in the tangential force component, as the abradable more easily gave way and pile up in front of the blade. The reduction in contact forces, particularly towards the final rubs was a result of the blade repeatedly being excited and vibrating at larger amplitudes before a steady state was achieved, making deeper than planned incursions. For both the compacted, and the aged and compacted abradable models at $200^\circ$C, the increased incursion depths due to the blade vibrations caused what would have been the final rubs fail to make contact. It is also important to note than no element deletion was employed to simulate abradable removal, and as such the contact forces seen in the model are higher than those that would be expected during experimental blade-casing rub testing [16], but does emphasise the influence that changing abradable conditions can have on the response of the blade.

## Conclusions

In this work, the influence of abradable pre-treatments on the quasi-static and high strain rate response over a range of temperatures has been investigated. These specimens were either in the as-sprayed state, thermally aged, compacted, or both thermally aged and compacted, and tests were conducted at room temperature, $110^\circ$C, or $200^\circ$C. Having a comprehensive understanding of the mechanical behaviour of abradables is crucial for the effective design and optimisation of compressors, such that the blade tip clearances in these systems can be further reduced by designing to tolerate blade-casing interactions. The key findings of this work are:

- A large temperature dependence was seen, with increases in failure strain seen at high and low strain rates with increasing temperature, though a significant reduction in failure stress was only seen under static conditions, or for the high-rate tests with compacted specimens.
- The abradable material exhibited a clear rate dependence. At lower strain rates, the mechanical response suggests that individual particles may compress and gradually realign to accommodate the applied load, whereas at higher strain rates, a more rapid reorientation is inferred. This behaviour is consistent with mechanisms reported in the literature for granular materials and is proposed here as a plausible explanation.
- The state of polyester phase was important, and had a large influence of the material response and failure behaviour. Ageing the abradable has been shown to increase the brittleness of the material. The aged polyester comprised more crystalline regions while amorphous regions were largely seen in the as-sprayed specimens, and thus has greater Young’s modulus and thermal stability, leading to an increase is failure stress of up to 10%. As a result, a greater load can be initially sustained until the bonds between particles break to accommodate realignment, but as a result can no longer carry and load. Whereas the amorphous polyester yields before debonding occurs, and therefore particle realignment can take place without debonding, though small loads can only be carried.
- By pre-compacting specimens thin AlSi struts are broken and the level of porosity was reduced [26]. These specimens saw a reduction in Young’s modulus with increasing temperature, as the stress was more uniformly distributed through both phases and became heavily dependant on the state of the polyester. By thermally ageing the compacted specimens, their failure stresses where increased by up to 50%.
- Using the experimentally determined abradable properties for a series of blade-casing rub models has shown a dependency of the blade response on the abradable condition and temperature. The first flap amplitudes seen when rubbing a compacted, or an aged and compacted abradable at $200^\circ$C were five times larger than interactions with an as-sprayed or aged abradable.

This work highlights the complexity of abradables, with the materials history having a substantial impact on its behaviour. By considering these factors, a deeper understanding of the compressive failure behaviour can be achieved, and used to better inform compressor blade and abradable system design. However, there is still a need to link the behaviours discussed in this work with the wear and adhesion mechanisms seen during blade-casing interactions.

## Data Availability

The data that support the findings of this study are not openly available due to reasons of sensitivity but may be available from the corresponding author upon reasonable request.
